# Residue-Based Thermogravimetric Analysis: A Novel Method to Quantify Carboxylate Group Modifications in Macromolecules

**DOI:** 10.1021/acs.biomac.5c01271

**Published:** 2025-10-17

**Authors:** Christos Leliopoulos, Hamidreza Mokhtari, Shima Tavakoli, Oommen P. Varghese

**Affiliations:** Translational Chemical Biology Laboratory, Division of Macromolecular Chemistry, Department of Chemistry-Ångström Laboratory, 99028 Uppsala University, Uppsala Se75121, Sweden

## Abstract

Quantifying the degree of modification (DoM) of hyaluronic acid (HA) is crucial for biomaterials development. This has remained a challenge, as diverse functional groups hinder precise spectroscopic quantification. Here, we present a method employing thermogravimetric analysis (TGA) by comparing residues of sodium hyaluronate (NaHA) and carboxylate-modified HA derivatives. Thermal decomposition enabled quantification of the inorganic residue (Na_2_CO_3_) that was obtained as the final product. Validation on four diverse HA derivatives, namely aldehyde, furan, thiol, and cyanoacetate, was performed. The first three matched ^1^H NMR/UV–vis data, while the cyanoacetate sample, previously unquantifiable, was determined for the first time. Because the residue arises solely from Na^+^, the assay is independent of the attached pendant group and potentially transferable to any carboxylate-bearing polymer beyond HA. Residue-based TGA closes an analytical gap, providing a label-free tool for quantifying carboxylate modification, applicable irrespective of chemical structure, and able to characterize “silent groups,” relevant for biomaterials.

## Graphical abstract

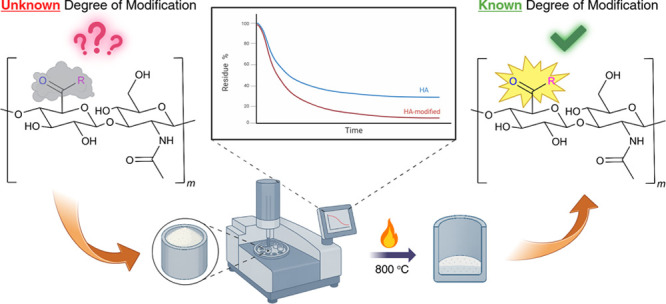

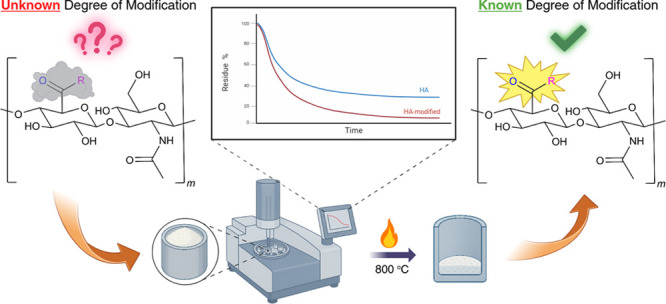

## 1 Introduction

Biomaterials derived from extracellular matrix (ECM)-based sources have been extensively explored to develop injectable fillers for esthetic treatments, as well as to fabricate advanced hydrogels for tissue engineering, regenerative medicine, and sophisticated drug-delivery systems.^1–3^ This broad range of applications highlights the urgent need for accurate, reliable, and straightforward methods to track chemical modifications in biomaterials, particularly driven by stringent regulatory requirements in medical device development, where precise documentation and rigorous control of material alterations are critical.^4,5^

Among the various ECM-derived biomaterials, hyaluronic acid (HA) has gained significant prominence because of its exceptional biocompatibility, safe biodegradability, and functional versatility.^6–8^ Naturally abundant in human ECM, HA is extensively employed in its sodium-salt form, sodium hyaluronate (NaHA), because of its superior water solubility, stability, rapid dissolution characteristics, and easier formulation processes compared to nonionized HA.^9^ The popularity of HA in biomedical applications largely stems from its ease of chemical modification, usually at the carboxylate end or the primary hydroxyls, allowing tailored adjustments to its physical and chemical properties.^10^ Such modifications are usually designed to facilitate cross-linking to obtain hydrogels or nanogels, or for bioconjugation purposes with drugs or other biologics.^11^ Site-specific modifications allow the fabrication of specific cross-linked structures, optimizing mechanical strength, improving biocompatibility, conjugating therapeutic agents, tuning degradation rates, enhancing processability, and controlling swelling behavior.^12^ HA, as a biomacromolecule for designing biomaterials, is particularly interesting owing to its precisely defined and stoichiometric structure, characterized consistently by a single carboxylate group per disaccharide repeat unit. This allows easy characterization of the modifications by ^1^H Nuclear Magnetic Resonance (NMR), provided the anticipated signals do not overlap with the characteristic protons of HA. Signals that are not visible spectroscopically present a challenge, and any ambiguity in determining the Degree of Modification (DoM) is crucial, as it can influence critical properties such as viscosity, cross-linking density, degradation kinetics, and overall biological performance. Consequently, precise determination of the DoM is of paramount importance.

Despite its significance, accurately quantifying the DoM in chemically modified HA remains a significant analytical challenge, and therefore, several strategies are sought. Traditional spectroscopic techniques, such as Fourier-transform infrared spectroscopy (FTIR), NMR, and UV–visible (UV–vis) spectroscopy, are frequently employed, but each presents inherent limitations. FTIR spectroscopy, while effective qualitatively in identifying chemical groups, generally lacks robust quantitative reliability due to challenges in baseline correction and signal integration. NMR spectroscopy excels for small-molecule analysis but faces considerable difficulties with large, complex polymers, where signal overlap or the DoM is below the detection threshold. Other challenges, such as broadened peaks, further complicate the spectra and limit quantitative accuracy and feasibility. Quantitative NMR analysis typically requires internal or external standards, which can introduce significant user variability and processing errors through baseline and integration errors. UV–vis spectroscopy offers rapid analysis but usually necessitates additional secondary labeling reactions (such as TNBS or Ellman’s assays) to detect specific functional groups, requiring cumbersome and error-prone calibration curves. Hydrophobic modifications of biopolymers often lead to self-assembly, limiting the anticipated *T*_2_ relaxation, affecting quantification by ^1^H NMR,^13^ or self-assembly-induced energy transfer, leading to lower UV or fluorescence signals.^2^ Stability and solubility issues of such modifications also exacerbate these problems, as solution-based analyses (NMR and UV–vis) become unreliable for nanoparticles or materials with poor solubility.

These collective limitations highlight a pronounced “quantification gap,” especially when analyzing complex polymers, solid-state materials, or hydrogels, where gel formation itself is often desired for the final application, yet makes postsynthesis characterization by solution-based methods virtually impossible. To comprehensively address these analytical challenges, we present a novel method employing thermogravimetric analysis (TGA) as an alternative approach capable of accurately quantifying chemical modifications in both gel and solid states, effectively bypassing the solubility and stability constraints of conventional spectroscopic techniques.

TGA, an established thermal-analysis technique widely used in polymer laboratories, precisely measures mass changes in materials subjected to controlled temperature or atmospheric conditions, providing valuable insights into thermal stability, decomposition kinetics, and compositional details. TGA has been extensively utilized for characterizing polymer blends, evaluating thermal degradation profiles, quantifying residual solvents or moisture critical for storage and processing, investigating additive effects (e.g., flame retardants and stabilizers), and assessing polymer purity. In polymer research specifically, TGA has proven to be crucial in elucidating dehydration and decomposition mechanisms and evaluating the impact of chemical modifications on thermal stability.

Historically, quantitative analyses using TGA have primarily employed two strategies: directly measuring mass loss attributed to additives or examining residual inorganic content postdecomposition.^14^ These complementary routes can be defined as “weigh what burns off” versus “weigh what is left.” Although promising, such a strategy has not been well explored to determine the DoM of biopolymers such as HA. Some examples of exploiting TGA as a method to quantify polymers include the quantification of grafted polystyrene on cellulose nanocrystals by identifying nonoverlapping decomposition temperatures specific to the polymer graft and comparing that with mass loss attributed to the grafted polymer, calibrated with the surface-polymer-initiator count in order to calculate the molar mass of each graft chain.^15^ Similarly, Lee and Bon accurately calculated polyethylene–glycol–methacrylate graft densities on graphene oxide using polymer-specific decomposition profiles and converted the loss into surface-normalized values of “chains nm^–2^,” after dividing by the known graphene oxide surface area.^16^ The most promising strategy was presented by Kaczmarska et al., where the inorganic residue was quantified and systematically correlated with degrees of substitution in sodium carboxymethyl-modified starch, showing that the residue dropped from ≈12% to ≈9% as the degree of substitution rose from 0.2 to 0.9, despite not developing it further into a quantification method.^17^ Similarly, the degree of substitution of acetylated starch was determined by TGA to measure the derivative of the decomposition, demonstrating that the degradation temperature increased with the degree of substitution.^18^

Building upon these precedents, we propose a novel TGA-based methodology to quantify chemical modifications in NaHA by measuring residue loss. In our approach, NaHA is completely oxidized at high temperatures, leaving behind a predictable residue (Na_2_CO_3_) that is directly proportional to the remaining free carboxylate groups. Thus, this residue measurement provides a straightforward and accurate quantification inversely related to the extent of modification. Our investigation covered four chemically modified HA derivatives: aldehyde, furan, thiol, and cyanoacetate groups, encompassing spectroscopically “visible” and spectroscopically “silent” scenarios. By rigorously comparing TGA-derived quantitative results against established NMR and UV-based analyses and employing unmodified HA as a control, we validate the accuracy, reliability, and broader applicability of TGA residue analysis for the precise determination of DoM. The side-by-side evaluation of spectroscopically “visible” and “silent” modifications further demonstrates TGA as an agnostic tool to determine the DoM where traditional methods such as FTIR, NMR, or UV–vis studies fail. We believe that our strategy has general applicability and could be applied to different types of macromolecules and even smaller molecules, potentially even those that do not have a well-defined structure.

## 2 Experimental Section

### 2.1 Materials

NaHA (molar mass of macromolecule: 200–400 kDa, purity: 97.9%, batch number: 837WTL) and 1-ethyl-3-(3-(dimethylamino)­propyl)-carbodiimide hydrochloride (EDC) were purchased from Glentham Life Sciences GmbH (Planegg, Germany). 1-Hydroxybenzotriazole hydrate (HOBt), 3-amino-1,2-propanediol, dithiothreitol (DTT), sodium periodate (NaIO_4_), sodium carbonate, sodium hydroxide (NaOH, purity: 99–100%, *K* ≤ 0.02%), furfurylamine (purity ≥ 99%), cyanoacetohydrazide (purity ≥ 98%), ethylene glycol (purity ≥ 99%), sodium acetate (CH_3_COONa, ≥99% purity), and other small molecules were obtained from Sigma-Aldrich (Steinheim, Germany). Pretreated dialysis membranes (Spectra/Por 7, molecular weight cutoff: 50 kDa) were purchased from VWR International AB (Kista, Stockholm, Sweden). The thiol-dihydrazide reagent was synthesized according to our previous protocol.^19^ All other chemicals were used without further purification unless otherwise noted. Deionized (DI) water was used throughout all the experiments.

### 2.2 Synthesis of HA Derivatives

#### 2.2.1 Synthesis of Aldehyde-Modified HA (HA-Ald)

HA-Ald was synthesized by following a published protocol. Briefly, HA (400 mg, 1 mmol, 1 equiv) was dissolved in 100 mL of DI water with HOBt (135 mg, 1 mmol, 1 equiv). 3-Amino-1,2-propanediol (91 mg, 1 mmol, 1 equiv) was added, and the pH was adjusted to 6.0. EDC (57 mg, 0.3 mmol, 0.3 equiv) was added in two portions at 30 min intervals, with stirring overnight. Dialysis against dilute HCl (pH 4.75, 0.1 M NaCl) was performed for 48 h (two times), followed by dialysis without NaCl for 24 h. Diol-modified HA was treated with NaIO_4_ (213 mg, 1 mmol, 1 equiv), quenched with ethylene glycol (310 mg, 5 mmol, 5 equiv), dialyzed against DI water (48 h), and lyophilized to yield HA-Ald. To quantify the aldehyde content by ^1^H NMR, the HA-Ald was reacted with *tert*-butyl carbazate, followed by reductive stabilization using NaCNBH_3_. The resulting product was subsequently dialyzed against pure deionized water for 24 h and lyophilized to remove any unreacted *tert*-butyl carbazate and NaCNBH_3_. The DoM of HA-Ald was determined by ^1^H NMR spectroscopy in D_2_O, following a previously reported protocol (Figure S4).^20^ The integration of the methyl signal at 1.98 ppm (assigned to the *N*-acetyl group native to HA) was normalized to 3. The methyl resonance of the conjugated *tert*-butyl group appeared at 1.42 ppm with an integration value of 0.91, which was used to determine the DoM to be 10.1%.

#### 2.2.2 Synthesis of Furan-Modified HA (HA-Furan)

HA-Furan was prepared via carbodiimide coupling chemistry. Briefly, HA (400 mg, 1 mmol, 1 equiv) and HOBt (135 mg, 1 mmol, 1 equiv) were dissolved in 100 mL of DI water. Furfurylamine (116 mg, 1 mmol, 1 equiv) was added and stirred until the solution became clear. The pH of the solution was adjusted to 5.5, followed by the addition of EDC (80 mg, 0.42 mmol, 0.42 equiv) in two portions. After overnight stirring at room temperature, the reaction mixture underwent dialysis against dilute HCl (pH 5, 0.1 M NaCl) for 24 h (three solvent changes) and subsequently against pure DI water for 48 h. The product was then lyophilized.

HA-Furan was characterized by ^1^H NMR spectroscopy in D_2_O (Figure S2). The spectra showed signals at 7.48, 6.43, and 6.38 ppm corresponding to protons of the furan ring, confirming the successful conjugation to HA.^21^ Additionally, by integrating these signals relative to the *N*-acetyl signal of native HA at 1.98 ppm, the DoM was calculated to be around 16%.

#### 2.2.3 Synthesis of Thiol-Modified HA (HA-Thiol)

Thiol-modified HA was synthesized following a previously reported protocol with some adjustments.^22^ Briefly, 400 mg of HA (1 mmol, 1 equiv with respect to disaccharide units) and HOBt (135 mg, 1 mmol, 1 equiv) were dissolved in 100 mL of DI water. Thiol-dihydrazide reagent (238 mg, 1 mmol, 1 equiv) was added and stirred until fully dissolved. The pH was adjusted to 4.5 using 1 M NaOH or HCl. EDC (67 mg, 0.35 mmol, 0.35 equiv) was subsequently added in two portions, maintaining the pH at 4.5. The reaction mixture was stirred overnight at room temperature. Then, DTT (700 mg, 4.5 mmol, 4.5 equiv) was added, and stirring continued overnight at room temperature. The product was purified by dialysis against dilute HCl (pH 5, 0.1 M NaCl) under nitrogen for 24 h (three solvent changes), followed by dialysis against dilute HCl (pH 5) for 24 h (four solvent changes), then with pure DI water for an additional 24 h, and lyophilized. The DoM for HA-Thiol was characterized by NMR and Ellman’s assay. The DoM for HA-Thiol was first determined by ^1^H NMR spectroscopy in D_2_O (Figure S3). Specifically, the signals corresponding to the methylene (−CH_2_CH_2_SH, 2.83 and 2.68 ppm) protons confirmed the successful conjugation of thiol groups to HA. The DoM was calculated by integrating the methylene signal relative to the *N*-acetyl peak of native HA (1.98 ppm), which serves as an internal standard. The DoM from NMR was 29.7%. Additionally, the DoM was further confirmed by Ellman’s assay employing the 5,5′-dithio-bis­(2-nitrobenzoic acid) reagent (DTNB). DTNB reacts with free thiols, resulting in the release of 5-thio-2-nitrobenzoic acid and the formation of a mixed disulfide.^23^ The concentration of free thiols in HA-Thiol was determined by measuring the absorption at 412 nm by using a UV–vis spectrometer. The DoM from Ellman’s assay was 30.1%.

#### 2.2.4 Synthesis of Cyano-Modified HA (HA-Cyano)

HA-Cyano was synthesized by hydrazide coupling. Briefly, HA (400 mg, 1 mmol, 1 equiv) was dissolved in 100 mL of DI water, followed by the addition of HOBt (135 mg, 1 mmol, 1 equiv). Cyanoacetohydrazide (99 mg, 1 mmol, 1 equiv) was added, stirred for 30 min, and the pH was adjusted to 4.75. EDC (76 mg, 0.4 mmol, 0.4 equiv) was added in three portions at 30 min intervals. After overnight stirring, dialysis was performed against dilute HCl (pH 5, 0.1 M NaCl) for 48 h, followed by dialysis in pure DI water for 24 h, and the product was lyophilized. The DoM for HA-Cyano was characterized using TGA due to the lack of distinct NMR or UV–vis signatures for the cyanoacetate functional group.

### 2.3 Sample Preparation Procedure for TGA Analysis

The unmodified HA and its derivatives (80 mg) were dissolved in 8 mL of DI water. The solution was filtered through a 0.45 μm poly­(vinylidene fluoride) or polyvinylidene difluoride (PVDF) syringe filter to remove particulates and contaminants. Syringes, needles, and filters were thoroughly washed with DI water to minimize contamination from manufacturing residues. The pH of the filtered solution was measured using a calibrated pH meter equipped with a microprobe and adjusted to a range of 7.5–8.0 using a freshly prepared 10 mM NaOH solution (0.4 g/L). The resulting solution was freeze-dried and then stored under a nitrogen atmosphere in sealed glass vials at −20 °C until analysis.

### 2.4 Procedure for TGA Analysis

Thermal analysis was conducted using a TGA/DSC 3+ instrument (Mettler Toledo AB, Stockholm, Sweden) equipped with an autosampler, an SDTA sensor, a large furnace, and an XP5U balance, suitable for crucibles and samples up to 900 μL and 5 g, respectively. Samples of ≈20 mg (range: 15–25 mg) were placed in alumina crucibles (300 μL capacity) fitted with lids to prevent sample loss. The 300 μL crucible was chosen so that the sample was not densely packed, thereby avoiding foaming from the decomposition gases. While an increased sample mass enhances the signal-to-noise ratio, it can also induce thermal gradients within the 300 μL pan. The influence of such thermal gradients is effectively compensated for by employing extended durations for the experimental segments. The instrument was calibrated using TGA-specific calibration weights (CarePac, class E2, Mettler Toledo). Drift and noise tests verified the instrument’s performance according to the manufacturer’s specifications. A sample weight at or above the USP-recommended minimum of 1.7 mg was targeted to minimize measurement error.

The TGA procedure included two sequential methods: a drying method and a thermal degradation and oxidative decomposition method. Balance equilibration for 5 min under nitrogen (50 mL/min) at 25 °C was performed before and after sample loading/unloading. Data were recorded at 1 s intervals. The heating rates and durations selected for the TGA experiments were chosen based on preliminary tests aimed at achieving optimal moisture removal, accurate baseline determination, and controlled decomposition conditions. Specifically, a heating rate of 15 °C/min was selected to balance time efficiency with the prevention of rapid volatilization, foaming, and uncontrolled combustion, while extended isothermal holding ensured thorough drying and complete oxidative decomposition. We have optimized and performed drying and analysis methods that were run in sequence, divided into four segments and six segments, respectively. The drying method, which consists of the 4 segments, was performed as follows:

**Segment 1:** Isothermal stabilization at 25 °C for 3 min under nitrogen.

**Segment 2:** Heating from 25 to 150 °C at 15 °C/min under nitrogen.

**Segment 3:** Isothermal hold at 150 °C for 200 min under nitrogen to effectively remove residual moisture.

**Segment 4:** Cooling to 25 °C and stabilization for 40 min under nitrogen.

**After the drying method was finalized, the thermal degradation and oxidative decomposition method was initiated for the determination of DoM as follows:**

**Segment 1:** Isothermal conditioning at 25 °C for 4 min under nitrogen, including a settling period of 6 min (bandwidth: 1 °C).

**Segment 2:** Heating from 25 to 150 °C at 15 °C/min under nitrogen.

**Segment 3:** Isothermal hold at 150 °C for 40 min under nitrogen.

**Segment 4:** Heating from 150 to 800 °C at 15 °C/min under nitrogen.

**Segment 5:** Isothermal hold at 800 °C for 120 min under air (50 mL/min) to facilitate the oxidative decomposition of carbonaceous residues. Na_2_CO_3_ starts to decompose into Na_2_O + CO_2_ above 850 °C; a safety margin of 50 °C was left to prevent this.

**Segment 6:** Cooling to 25 °C and stabilization for 40 min under nitrogen.

Blank measurements were performed four times to obtain a mean blank curve. The mean blank curve was subtracted from the sample data to correct buoyancy, drift effects, and other instrumental artifacts.^24^

### 2.5 Characterization of TGA Residues

Residues from TGA were characterized by using FTIR and scanning electron microscopy coupled with energy-dispersive X-ray spectroscopy (SEM-EDX).

For FTIR analysis, spectra were recorded with an IRTracer-100 spectrometer (Shimadzu), scanning from 400 to 4000 cm^–1^ at a resolution of 4 cm^–1^, with 45 scans per sample. Residues were analyzed as powders obtained by scraping crucibles. Commercially available Na_2_CO_3_ served as a reference to verify the residue composition, while commercially available NaCl and NaOH were used as controls.

For SEM-EDX analysis, the presence of Na^+^ within the residues and their corresponding micromorphology were investigated by Scanning Electron Microscopy coupled with energy-dispersive X-ray spectroscopy (SEM-EDX). A Zeiss Merlin Field Emission Gun Scanning Electron Microscope (FEG-SEM), operated at an accelerating voltage of 10 kV, was utilized for these analyses. Samples were analyzed using standard operating protocols for SEM imaging and elemental composition analysis. The samples for SEM and EDX analysis were prepared by placing residue particles onto double-sided conductive carbon tape. To prevent charging effects during imaging, a thin gold coating (∼5 nm) was sputtered onto the sample surface prior to analysis. The surfaces and cross-sections of the samples were examined using a field emission scanning electron microscope (FEG-SEM, Zeiss Merlin). For imaging, secondary electron (SE) detectors, specifically in-lens annular-type detectors, were employed to capture high-resolution surface morphology. Elemental analysis and X-ray mapping were conducted using the same SEM equipped with an X-Max 80 mm^2^ Silicon Drift EDX Detector (Oxford Instruments), which has high sensitivity for analysis at elevated count rates. Data acquisition and analysis were performed using Oxford AZtec software. Elemental mapping was performed to assess the distribution of elements across the sample and quantification was achieved through point analysis and spectral fitting.

## 3 Results and Discussion

Novel strategies to determine the DoM are imperative, as current methods have inherent limitations. Specific cases illustrate these limitations clearly. Aldehyde-modified HA usually shows weak ^1^H NMR signals, and aliphatic aldehydes often become hydrated to yield geminal diols.^25^ Therefore, we and others have relied on secondary labeling methods with nucleophilic reagents, such as *tert*-butyl carbazate, to yield products that are “visible” by ^1^H NMR. This, however, requires proper purification of the products before quantification by NMR and can easily generate false positives (unreacted label) or false negatives (incomplete conversion). We have previously reported an alternative method for aldehyde quantification by determining the amount of periodate consumed during the oxidation of strategically incorporated vicinal diols to prevent oxidation of the sugar rings.^20^ Thiol derivatives, on the other hand, have inherent problems with oxidation in the presence of molecular oxygen, which invariably affects UV–vis-based colorimetric assays such as Ellman’s assay. We have previously attempted to solve this problem by carefully degassing the solvent before reducing any disulfides, followed by maintaining an acidic pH that prevents oxidation.^19^ This strategy is quite tricky and requires careful experimentation to obtain reproducible results. We also designed a novel HA derivative having cyanoacetate modifications that lack distinct ^1^H NMR or UV signals altogether, posing a severe quantification challenge.

### 3.1 Synthesis of HA Derivatives

To evaluate a residue-based approach for quantifying carboxylate substitution, we synthesized various chemically modified NaHA, namely HA derivatives having aldehyde-, furan-, thiol-, and cyanoacetate modifications, denoted as HA-Ald, HA-Furan, HA-Thiol, and HA-Cyano, respectively. We employed carbodiimide chemistry to conjugate different functional groups, specifically at pH 4.7 for hydrazide-modified reagents^23^ and pH 6 for amine-modified reagents (Scheme 1).^20^ Characterization of these derivatives was carried out using conventional techniques as described earlier. HA-Furan and HA-Thiol each displayed a unique NMR resonance that allows the classical determination of the DoM, whereas HA-Ald could be quantified only after an additional derivatization reaction. The DoM of HA-Thiol was also measured with Ellman’s assay using UV–vis. However, as discussed above, HA-Cyano lacks any validated analytical protocol, representing a spectroscopically “silent” modification.

**Scheme 1:**
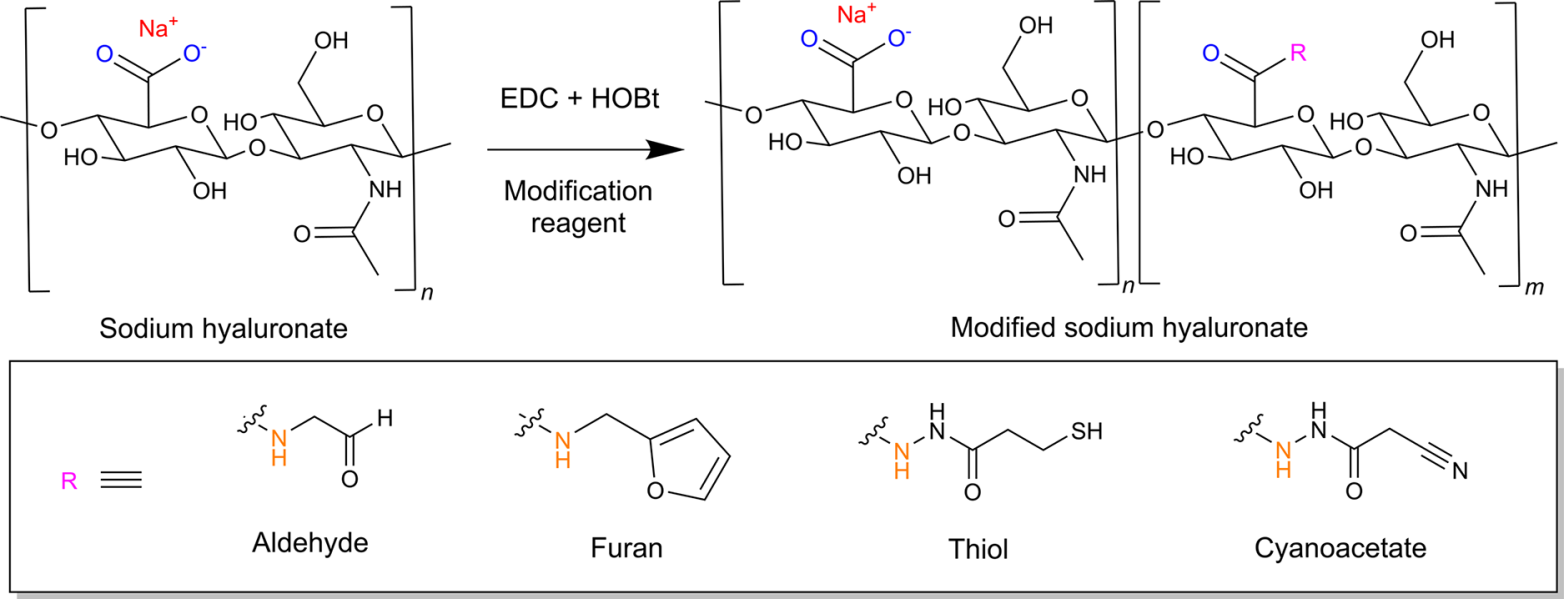
Schematic Representation of NaHA and Its Chemical Modifications^a^

a The native NaHA disaccharide unit contains sodium carboxylate groups that serve as reactive sites for derivatization. HA derivatives were synthesized using coupling carbodiimide chemistry to obtain aldehyde, furan, thiol, and cyanoacetate groups that represent spectroscopically visible and silent modifications, as shown.

### 3.2 TGA as a Method to Determine DoM of HA Derivatives

To accurately quantify the extent of chemical modification in HA, we leveraged a fundamental property inherent to carboxylic acids’ tendency to form carboxylate ions under elevated pH conditions. These negatively charged carboxylate ions readily establish ionic interactions, known as salt bridges, with various cations. Commercially, Na^+^ is the predominantly used counterions for HA formulations, although K^+^, Ca^2+^, and Mg^2+^ can also be used, as they can form stable electrostatic associations with the carboxylate groups.

When NaHA is heated to high temperatures in an oxidizing environment, it decomposes completely and leaves behind a predictable amount of Na_2_CO_3_. Each carboxylate that is chemically converted into various derivatives through different reactions (e.g., amide or hydrazide chemistries) does not produce Na_2_CO_3_ upon decomposition, paving the way to accurately determine the amount of residue obtained, which directly reflects the number of free carboxylates available in the biopolymer and, hence, the DoM within the biopolymer.

The selection of an appropriate counterion significantly influences the physicochemical behavior of HA. Na^+^ exhibits a notably higher affinity for carboxylate groups compared to K^+^, primarily due to Na’s^+^ smaller ionic radius and greater charge density, which enhance the stability of the ionic interactions. Additionally, Na^+^ is monovalent, in contrast to divalent Ca^2+^ and Mg^2+^. Divalent ions can establish ionic cross-links within the polymeric structure, which, while potentially beneficial in certain controlled environments, typically lead to undesirable consequences such as polymer precipitation, excessive physical cross-linking, and reduced processability.

Moreover, practical considerations further discourage the use of Ca^2+^ in the development of HA-based formulations. Ca­(OH)_2_ exhibits limited solubility in DI water and readily forms precipitates of CaCO_3_ upon exposure to atmospheric carbon dioxide (CO_2_). DI water, when exposed to atmospheric CO_2_, establishes an equilibrium involving carbonic acid (H_2_CO_3_), bicarbonate (HCO_3_^–^), and carbonate (CO_3_^2–^) species, depending on the solution pH. Such precipitation phenomena complicate handling, processing, and formulation consistency, reinforcing the preference for Na^+^ as the counterion of choice.

The intrinsic acidity of HA, characterized by a p*K*_a_ of approximately 3.2,^26^ underscores the relevance of precise pH control. The extent of dissociation can be estimated from the Henderson–Hasselbalch relationship (eqs S1–S3).

As illustrated in Figure 1A and Table S1, at pH 3.2, HA exhibits 50% ionization, whereas at physiological and higher pH levels (≥7.2), ionization dramatically increases to over 99.99%.

**Figure 1:**
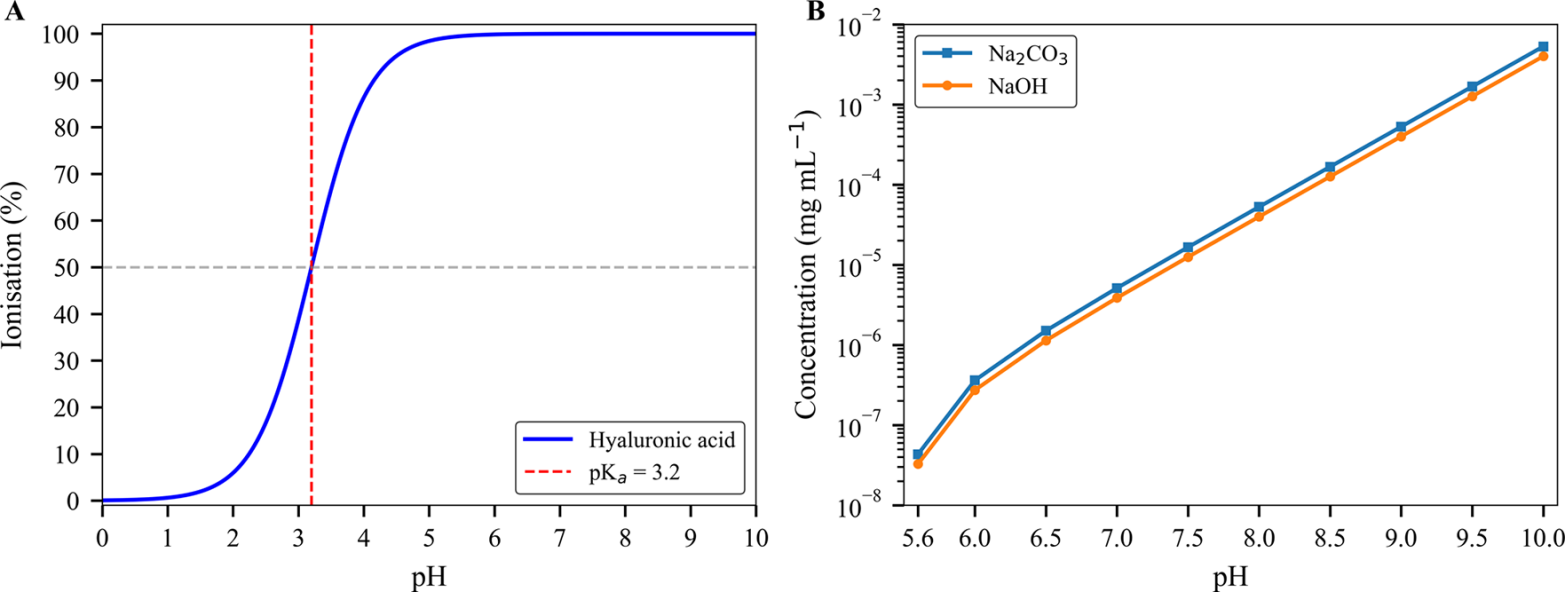
Ionization behavior, buffer capacity, and TGA methodological framework. (A) Theoretical ionization profile of the carboxyl functional group in HA as a function of pH, modeled using the Henderson–Hasselbalch equation with a p*K*_a_ of 3.2. This curve illustrates the extent of ionization (% deprotonation) across physiologically relevant pH values. (B) Log-scale plot of the NaOH concentration required to raise the pH from an initial value of 5.6 (typical of DI water) to progressively higher values. For comparison, the theoretical concentration of Na_2_CO_3_ is also plotted, assuming complete conversion of all Na^+^ to Na_2_CO_3_.

Figure 1B and Table S2 further demonstrate the quantitative relationship between pH adjustment and the NaOH used for this purpose. Due to the logarithmic scale of pH, each incremental increase corresponds roughly to a 10-fold rise in the amount of NaOH necessary per unit volume. The natural equilibrium of DI water with atmospheric CO_2_ typically stabilizes around a mildly acidic pH of 5.6.^27^ Under acidic conditions, dissolved CO_2_ predominantly exists in equilibrium with H_2_CO_3_. However, as pH increases to alkaline conditions, the equilibrium progressively shifts toward bicarbonate and carbonate ions, contingent on Na^+^ availability, with the latter two compounds being solids once the water is removed.

During the chemical modification and purification of HA, the polymer typically maintains a subneutral pH (below pH 7), indicating incomplete conversion of the polymer to its Na^+^ salt form. Thus, to achieve complete ionization and improve material consistency, supplemental Na^+^ is systematically introduced into the system through controlled titration with NaOH. Given the p*K*_a_ of HA being ∼3.2, an observable inflection point in pH titration is expected near this acidic threshold. The targeted pH range of 7.5–8.0 was strategically chosen to maximize conversion efficiency while minimizing unintended artifacts from mass increase contributions from Na_2_CO_3_ formation. At pH 7.5, the Na_2_CO_3_ concentration in a solution of DI water is approximately 1.66 × 10^–5^ mg/mL, and at pH 8.0, it rises slightly to around 5.28 × 10^–5^ mg/mL, assuming full conversion of all available free-floating Na^+^ to Na_2_CO_3_. These pH values effectively facilitate nearly complete (≥99.99%) conversion to NaHA without significantly impacting experimental precision or accuracy related to the characterization of bound Na^+^ due to the minuscule and undetectable mass of Na_2_CO_3_ at these concentrations, assuming the final solution volume remains low (less than 10 mL). The reason behind this argument is that the free Na^+^ is substantially lower in concentration compared to the grafted Na^+^ on the HA molecule and thus can be effectively ignored. This careful balance ensures optimal modification and stabilization of HA, supporting its widespread practical application in biomedical and pharmaceutical products.

### 3.3 TGA Protocol and Blank Stability

In the current study, a mean blank curve was meticulously determined from four replicate measurements (*n* = 4). This blank curve, along with the experimental TGA methodology, is presented in Figure 2. The instrument error, calculated as the standard deviation of these four blank measurements, was found to be exceptionally low, typically less than ±0.001 mg. For the recommended minimum sample mass of 1.7 mg, this error corresponds to a relative measurement uncertainty of approximately 0.059%, thereby underscoring the robustness of the employed analytical method.

**Figure 2:**
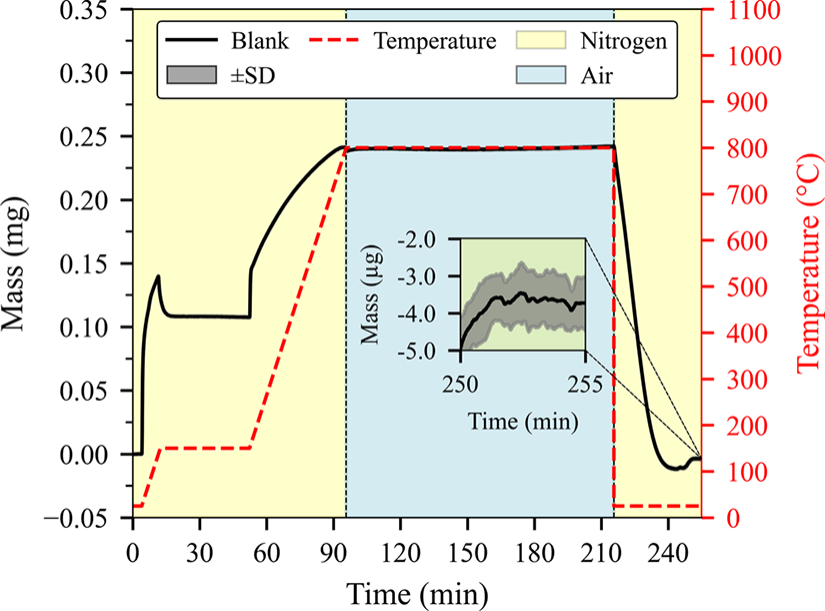
Experimental TGA procedure showing the temperature profile (dashed red line), gas atmosphere phases (nitrogen and air), and blank sample mass change over time. The inset highlights the final segment of the TGA curve (*n* = 4 replicates) with an associated envelope standard deviation (SD) of ±0.001 mg, ensuring a high-resolution assessment of baseline signal stability.

To obtain quantitative TGA data, we programmed a sequence of drying followed by six-segment thermal-degradation TGA methods that were carried out under carefully controlled gas atmospheres. Before Segment 1 is initiated, the balance is equilibrated at room temperature under the same nitrogen atmosphere that will be used during the run and then tared. The empty, lidded crucible is placed on the pan and allowed to re-equilibrate, and its mass is recorded. The crucible is removed, loaded with the sample, and returned to the balance, and after a second equilibration period, tare Segment 1 begins. The sample weight is recorded, and the furnace is held isothermally to provide a final balance-stabilization period before any programmed heating. Segment 2 then heats the sample to 150 °C, and Segment 3 maintains this temperature to guarantee complete removal of moisture. Segment 4 ramps the temperature to 800 °C at 15 °C/min under nitrogen. The slow, inert ramp, the large crucible volume, and the lid prevent violent combustion, gas entrapment, sample ejection, or foaming. By the end of this stage, the organic content is completely degraded, leaving organic and inorganic residue. Segment 5 introduces air into the system while keeping the temperature at 800 °C, promoting combustion of residual char and yielding only inorganic residue. The temperature of 800 °C was chosen because it is significantly above the organic decomposition temperature and still below the Na_2_CO_3_ melting temperature. Finally, in Segment 6, the atmosphere reverts to nitrogen, and the system cools to 25 °C. Nitrogen prevents moisture absorption and artificial weight gain, while cooling to 25 °C minimizes buoyancy effects that could otherwise introduce errors during blank subtraction at higher temperatures.

### 3.4 Calibration of Native HA to Optimize TGA Method for Quantification

One of the challenges of using TGA as a method for quantification includes inherent organic or inorganic impurities that can affect quantification. We therefore decided to first determine the experimental carboxylate residue and compare it with anticipated values from a theoretically 100% HA. Of note, most commercially available NaHA has 5–10% impurities that need to be calibrated to remove systematic measurement errors. For the quantification, we adjusted the pH of a small-volume solution to obtain a target pH range of 7.5 to 8.0 for the HA solution, ensuring a carboxylic acid ionization degree above 99.995% and minimizing the concentration of free Na^+^. To evaluate reproducibility across different sample sizes, we analyzed varying masses (15 mg, 20 mg, and 25 mg) of unmodified NaHA. As illustrated in Figure 3, the measurements showed excellent correlation, prompting us to calculate a mean residue from these nine measurements, resulting in an average residue of 12.56% for the unmodified NaHA. The residue of different sodium-containing organic molecules has been consistently identified as Na_2_CO_3_ in existing literature.^28^

**Figure 3:**
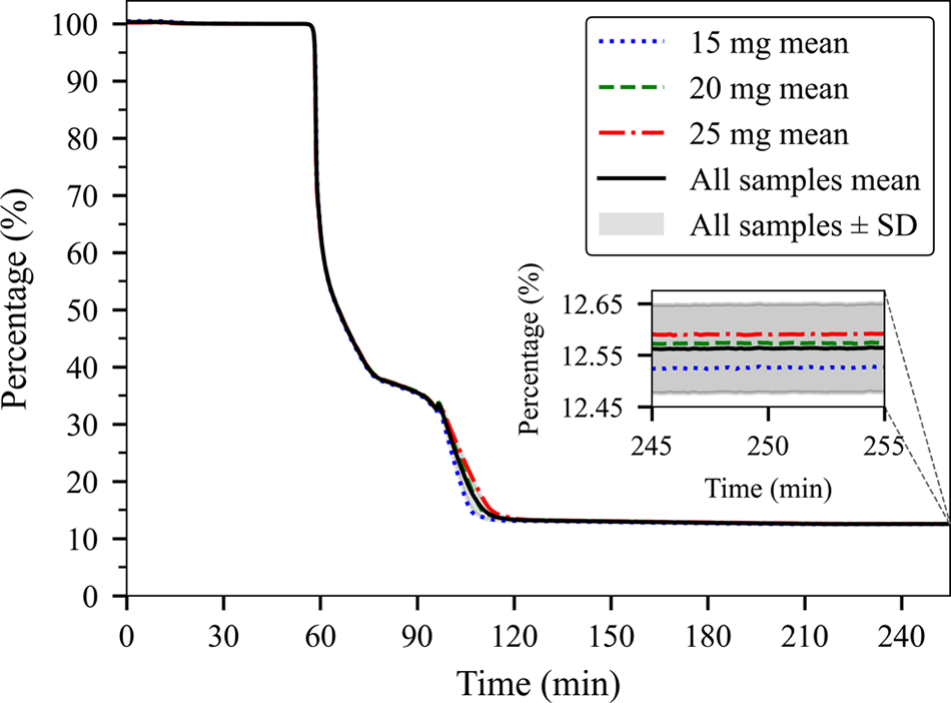
TGA of unmodified pH-adjusted NaHA representing percentage mass loss curves for three different initial sample masses (15, 20, and 25 mg), *n* = 3 for each, showing excellent reproducibility. The final residue plateau is enlarged in the inset to highlight the minimal variance between samples and confirm consistent TGA performance.

To experimentally confirm the identity of the residue, we performed a series of analytical techniques (Figure 4). To confirm the residue, we first performed scanning electron microscopy (SEM) coupled with SEM-EDX. SEM images of the TGA residue (Figure 4A), elemental mapping (Figure 4B), and quantitative elemental analysis (Figure 4C) collectively revealed a Na^+^ content of approximately 99.2%. It should be noted that routine EDX cannot reliably quantify carbon or oxygen, and therefore the expected CO_3_^2–^ partners are not represented in this measurement. Additionally, we conducted FTIR analyses, as displayed in Figure 4D, and compared the residue with pure Na_2_CO_3_ powder. Thus, this validates the residue as nearly pure Na_2_CO_3_. The presence of carbon, aluminum, and gold in the EDX spectra (Figure 4C) is from the carbon tape, the aluminum sample holder/stage, and the gold coating, respectively.

**Figure 4:**
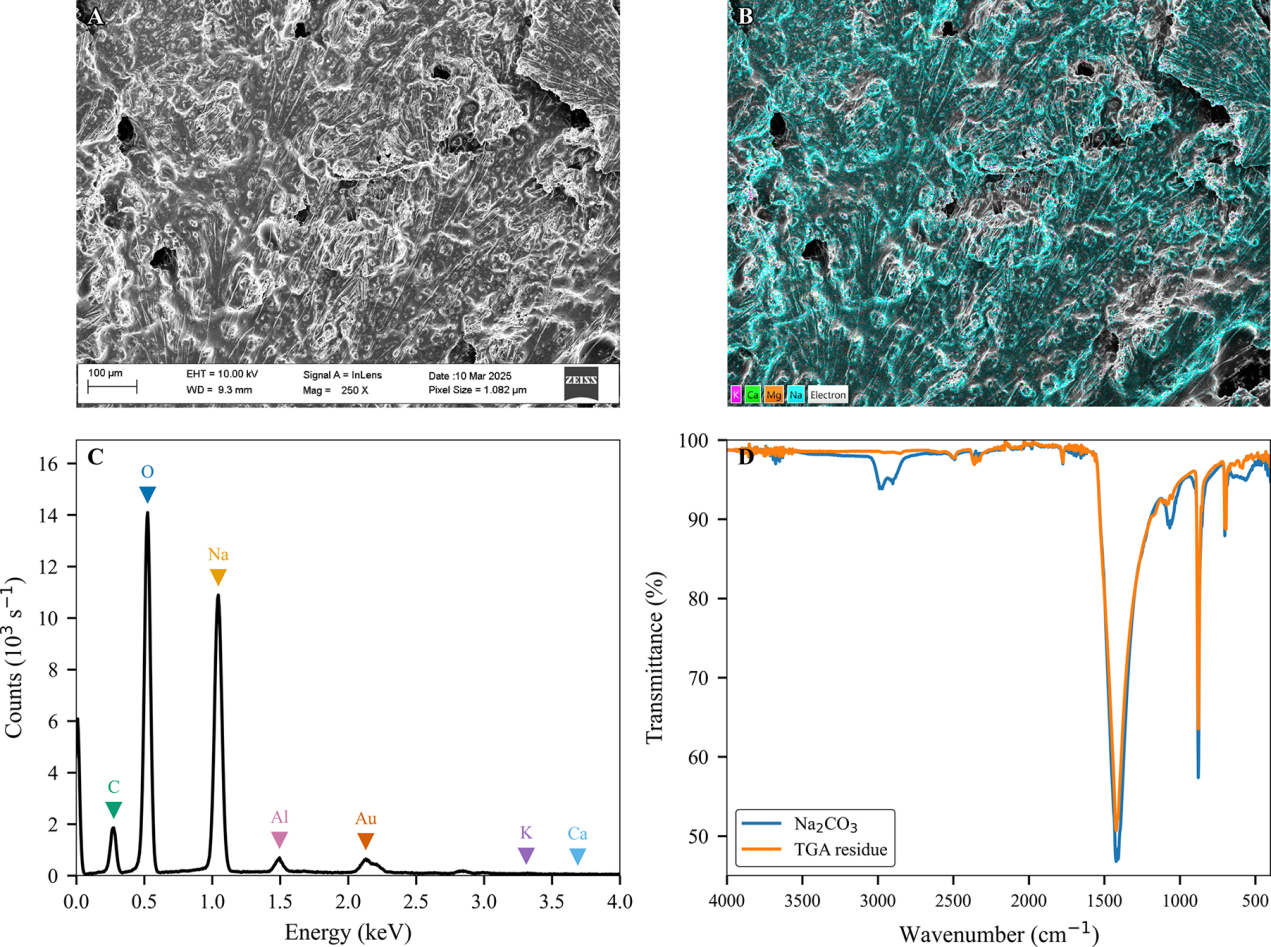
Characterization of Na_2_CO_3_ in the TGA residue. (A) SEM image showing the surface morphology of the sample after thermal degradation, with visible inorganic crystalline structures. (B) EDX elemental mapping highlighting the distribution of Na^+^ (cyan), confirming the widespread presence of Na_2_CO_3_. (C) EDX spectrum of the residue showing major elemental peaks for Na^+^, consistent with the composition of Na_2_CO_3_. (D) FTIR spectra comparing commercial Na_2_CO_3_ with the TGA residue, showing matching characteristic peaks and confirming the identity of the residue as Na_2_CO_3_.

The FT-IR comparison (Figure 4D) shows that, across the 4000–500 cm^–1^ region, the spectrum of the TGA residue (orange) is virtually superimposable on the sodium carbonate reference (blue). Such complete overlap is the qualitative hallmark indicating that both traces correspond to the same carbonate compound.

Elemental analysis by SEM-EDX further corroborates that the residue is a sodium-rich carbonate phase. The elemental map (Figure 4B) is dominated by Na^+^ (rendered cyan), and the quantitative spectrum (Figure 4C) attributes ≈99 wt % of the detected signal to Na^+^, with only trace K^+^ and no detectable Ca^2+^ or Mg^2+^. Because EDX cannot quantify carbon or oxygen reliably, the expected CO_3_^2–^ ions are invisible. Nevertheless, the absence of Ca^2+^ rules out CaCO_3_, and the lack of Cl^–^ eliminates NaCl. We therefore believe that Na_2_CO_3_ is the only composition consistent with the observed elemental distribution, allowing us to quantitatively determine the DoM.

We have also performed our optimized TGA procedure on a sample of commercially available CH_3_COONa (Figure S1), which is expected to produce a theoretical Na_2_CO_3_ of 64.6%, by using eq S2 and adjusting it for Na_2_CO_3_ instead of NaHA with eq S1. Under our conditions, we observed a residue of 63.97% ± 0.18%, which corresponds to 1% lower than the theoretical value. This result can be attributed to the CH_3_COONa purity of 99%. This stoichiometric agreement, together with the EDX and FTIR measurements in Figure 4B,C,D strongly suggests that the TGA residue is Na_2_CO_3_ and also showcases that this TGA procedure can be used to quantify the DoM of carboxylate groups or potentially other negatively ionizable moieties, for small or macromolecules alike. Commercial NaCl and NaOH were also measured by FTIR as reference sodium salts (Figure S5).

To theoretically validate our experimental residue data, we used the following equation:

1 $$R_{Th}=\frac{M_{Na_{2}CO_{3}}}{2\times M_{NaHA}}$$

which can be rearranged to determine the theoretical residue of Na_2_CO_3_ for NaHA:

$$R_{\mathrm{Th}}\left(\%\right)=\frac{52.99}{401.3}\times 100$$

$$R_{Th}\left(\%\right)=13.21\%$$

Here, *R*_Th_ and *R*_Th_ (%) represent the theoretical residue fraction and percentage for pure NaHA, respectively, $M_{Na_{2}CO_{3}}$ is the molar mass of Na_2_CO_3_ and *M*_NaHA_ is the molar mass of the repeating unit of NaHA. The factor of 2 accounts for the molar ratio, reflecting that one molecule of Na_2_CO_3_ contains two Na^+^ corresponding to two carboxylate groups from two NaHA repeating units.

The small difference between the theoretical (13.21%) and experimental (12.56%) residues is hypothesized to reflect impurities in the commercial NaHA.^29^ Following our in-house preparation and 50 kDa dialysis, the purity of the starting material can shift from the manufacturer’s reported value: low molar mass impurities (and short HA chains) will pass through the membrane, whereas additional high molar mass non-HA species may be retained above the cutoff. The latter increase the predecomposition mass yet are not part of NaHA and do not contribute to Na_2_CO_3_ formation, decreasing the residue %, while the former reduce the mass attributable to HA chains, increasing the residue %. Together, these effects can drive the measured residue upward or downward relative to the reported purity value. For scientific rigor, we adopt an internal standard and report the residue determined experimentally in this study (12.56%). Hence, we recalculated the average molar mass per carboxylate ion using the experimentally obtained residue using the following equation:

2 $$M_{COONa}=\frac{M_{Na_{2}CO_{3}}}{{2\times R}_{Exp}}$$

This corresponds as follows for unmodified NaHA:

$$M_{COONa}=\frac{52.99}{0.1256}$$

$$M_{COONa}=421.93\;g/\mathrm{mol}$$

Here, *M*_COONa_ is the average molar mass per carboxylate ion, and *R*_EXP_ and $R_{Exp}\;(\%)$ is the real TGA residue expressed as both a fraction and a percentage, as observed experimentally. By application of eq 2, which incorporates a correction for the true residue, a revised average molar mass of 421.93 g/mol per carboxylate moiety was calculated for the control NaHA sample. This precise determination of *M*_COONa_ is critical for accurate calibration. Consequently, it is recommended that each new batch of NaHA be characterized to establish its specific experimental average molar mass per carboxylate ion, ensuring reliable and consistent data.

With the revised average molar mass per carboxylate ion (421.93 g/mol), we can now accurately calculate the DoM in the chemically altered NaHA samples. When HA undergoes chemical modification, specifically at the carboxylate residue, it effectively reduces the number of carboxylate binding sites available to Na^+^. Consequently, during TGA, the modified samples yield a lower Na_2_CO_3_ residue compared to the unmodified form, as fewer Na^+^ molecules are bound to the polymer backbone. This direct relationship means that a higher DoM corresponds to fewer available carboxylate groups, thus resulting in fewer Na_2_CO_3_ residues. Equation 3 explicitly demonstrates this relationship, quantitatively linking the residue content to the extent of chemical modification.

3 $$R_{\mathrm{Exp}}=\frac{\left(1-DoM_{r}\right)\times \frac{M_{{\mathrm{Na}}_{2}{\mathrm{CO}}_{3}}}{2}}{DoM_{r}\times M_{\mathrm{Sub}}+M_{\mathrm{COONa}}}$$

Here, DoM_*r*_ is the fraction of the degree of modification, and *M*_Sub_ is the difference in molar mass accounting for the weight increase introduced by the substitution. In the case of HA-Furan, the polymer was modified with the small molecule furfurylamine, which has a molar mass of 97.11 g/mol. We need to account for the chemical changes occurring during the modification process. Specifically, each modification reaction involves removing one Na^+^ (22.98 g/mol) originally bound to the carboxylate group of the NaHA and one H^+^ (1.01 g/mol) from the amino group of the furfurylamine. After these masses are subtracted, the net increase in the molar mass of each modified repeat unit of NaHA is calculated to be 73.12 g/mol, resulting in a final molar mass of the modified repeat unit of 474.42 g/mol. This incremental increase reflects the precise structural changes in the polymer, directly resulting from the addition of furfurylamine and the removal of the corresponding Na^+^ and H^+^.

The DoM is calculated by rearranging eqs 3 and 4:

4 $$DoM_{r}=\frac{\frac{M_{Na_{2}CO_{3}}}{2}-R_{Exp}\times M_{COONa}}{\frac{M_{Na_{2}CO_{3}}}{2}+R_{Exp}\times M_{Sub}}$$

Using the experimentally determined residue of 10.28% for HA-Furan and substituting the values for eq 4:

$$\mathrm{DoM}\left(\%\right)=\frac{52.99-0.1028\times 421.93}{0.1028\times 73.12+52.99}\times 100=15.85\%$$

DoM(%)=15.85%

where DoM(%) is the degree of modification represented as a percentage. This yields a DoM for a single measurement of 15.85% for HA-Furan. Given this, we can calculate the DoM for the rest of the modified HA derivatives (HA-Ald, HA-Thiol, and HA-Cyano) using the values obtained from the TGA measurement, as represented in Table S4. The data are plotted in Figure 5 and compared with the experimental data acquired from ^1^H NMR.

**Figure 5:**
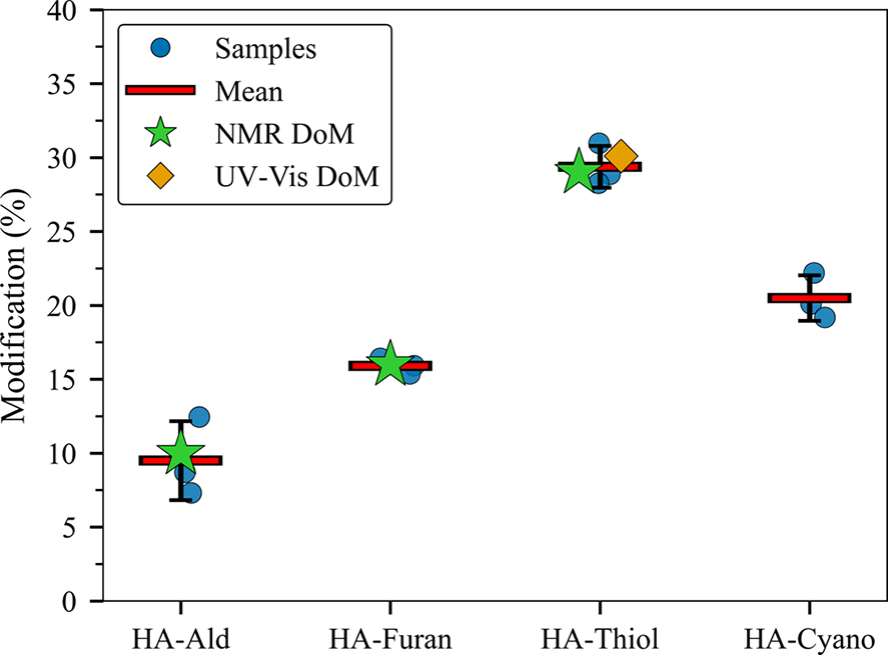
DoM for four chemically modified HA derivatives, namely, aldehyde, furan, thiol, and cyanoacetate, as determined by TGA. Blue circles show individual TGA replicates (*n* = 3); red horizontal bars with black whiskers indicate the mean ± SD. Green stars mark reference DoM values obtained by ^1^H NMR for every derivative, while the orange diamond denotes the UV–vis DoM value available for the HA-Thiol sample. TGA estimates correspond to mass loss attributable to carboxylate substitution, whereas the spectroscopic methods provide a molecular-level confirmation of substitution efficiency.

With the revised calculations, the DoM obtained from TGA for HA-Furan is 15.91%, closely matching the 16% determined by ^1^H NMR. The same correlation is observed for HA-Ald (TGA = 9.49%, NMR = 10.1%) and HA-Thiol (TGA = 29.38%, NMR = 29.7%, UV–vis = 30.1%), as summarized in Table S3, confirming that the TGA protocol yields quantitative data that agree well with the conventional NMR approach.

By contrast, in the HA-Cyano case, TGA assigns a DoM of 20.5%, but no reliable ^1^H NMR (or UV–vis) value can be quoted. The cyanoacetate pendant group contains only aliphatic protons, whose resonances merge with the broad carbohydrate envelope of the HA backbone. The diagnostic C≡N functionality is silent in ^1^H NMR and gives a very weak ^13^C signal at the low substitution levels employed. Moreover, the isolated nitrile does not introduce a distinct chromophore in the UV–Vis window (>200 nm), so absorbance-based quantification by UV–vis spectroscopy is also impossible, unless a dedicated cyano-specific labeling strategy is adopted, analogous to the aldehyde labeling method already validated for HA-Ald. Consequently, TGA remains the only practical tool for determining the DoM of HA-Cyano, underscoring the utility of the present thermal method for modifications that escape both NMR and UV–vis detection.

### 3.5 Limitations and Considerations

It is worth mentioning that the DoM corresponds to the carboxylate groups’ DoM and not any other form of modification (e.g., hydroxyl groups) in the HA case study. This protocol could be potentially modified to fit other negatively charged moieties, organic molecules, or counterions that could lead to a different residue.

Additionally, since eq 4 is not applicable to molecules or macromolecules with unknown structures but that still possess negatively charged moieties, an alternative, simplified equation can be used (Eq S1) to calculate the DoM. The value of *M*_sub_ found in eq 4, if used to quantify an unknown compound, should be close to the molar mass of Na^+^ (23 g/mol) if Na^+^ is the expected counterion. The DoM error becomes greater as these numbers deviate. Consequently, the TGA assay cannot be used to fully characterize the chemical structure or the identity of the pendant group, and other complementary methods should be utilized.

Reliable application of this TGA-based protocol hinges primarily on minimizing extraneous inorganic matter. Environmental dust or unintended salts can adsorb onto the HA sample and carry through to the final ash, artificially inflating the residue or the sample mass. To suppress this risk, we used ion-free, pretreated dialysis membranes and ultrapure NaOH for every pH adjustment and filtered each solution immediately before freeze-drying. These precautions increase the likelihood that the residue truly originates from the intended modification rather than from external contamination. To minimize ionic interaction artifacts in determining DoM, cationic modifications (e.g., amino-modified macromolecules) should be extensively dialyzed at pH 5 with 0.1 M NaCl to disrupt electrostatic interactions. To remove excess salt, they should be further dialyzed in deionized water.

A second source of error is sodium introduced during sample preparation. Even with ultrapure reagents, the Na^+^ required for pH control can convert dissolved CO_2_ into additional Na_2_CO_3_, raising the ash mass and thereby underestimating the DoM. We therefore monitored pH with a freshly calibrated pH meter and freshly prepared NaOH, adjusted with the minimum base required to stay within the target range, and kept the final solution volume below 10 mL to limit “free” carbonate formation. Thermal decompositions could potentially give other products that could likely change the expected residue %, while in our study we mainly observed Na_2_CO_3_ under our experimental conditions.^30^

Finally, the instrument’s performance sets the floor for quantitative accuracy. A certified, well-maintained TGA was used, external reference weights were applied regularly, and the balance was allowed to reach full equilibrium before each tare and every crucible or sample weighing. Even milligram-scale drift at this stage propagates into sizable errors in calculated DoM, underscoring the need for rigorous calibration and balance-stabilization protocols alongside the chemical precautions described above.

## 4 Conclusion

This work presents a residue-based TGA protocol that quantifies chemical modification in carboxylate-bearing polysaccharides simply by weighing what remains after complete thermal oxidation. By converting HA to its sodium salt, correlating the residual Na_2_CO_3_ mass with unreacted carboxylate groups, and performing milligram-scale TGA runs followed by blank subtraction, the method transforms the long-standing challenge of “measuring what is added” into the easier and modification-agnostic task of “measuring what is lost.”

When applied to four structurally diverse HA derivatives—namely aldehyde, furan, thiol, and the “spectroscopically silent” cyanoacetate—the protocol yielded mean DoM values that deviated by <1% from the values obtained by ^1^H NMR, while maintaining an instrumental uncertainty of ≤0.06%. Because the workflow needs only one pH-adjustment step and minimal sample, single-run measurements are justified when material is scarce and sample preparation is not easy.

Although demonstrated with HA, the approach can be inherently transferred to any carboxylate-containing polymer formulated as a Na^+^ salt. Its simplicity, label-free operation, and subpercent precision address key regulatory concerns of lot-to-lot consistency, method traceability, and suitability for gels or solids, without relying on complementary spectroscopic assays. Residue-based TGA thus offers a robust, universally accessible tool for the quantitative characterization of chemically modified polysaccharides in both biomedical and industrial settings, closing a critical analytical gap and accelerating product development and standardization.

## Supplementary Material



## Notes

Supplementary equations; TGA on sodium acetate as per the TGA procedure described; NMR characterization of HA-Furan, HA-Thiol, and HA-Ald; FTIR measurement of reference compounds (NaCl, NaOH, and Na_2_CO_3_); table with ionization percentage of hyaluronic acid at different pH values; table with pH value of different NaOH concentrations and the conversion to Na_2_CO_3_ concentration and a table with compilation of DoM from all methods for different HA derivatives (PDF)
