# Supplementary material for: Residue-Based Thermogravimetric Analysis: A Novel Method to Quantify Carboxylate Group Modifications in Macromolecules
Source: Biomacromolecules. 2025 Oct 17;26(11):7767–77. doi: 10.1021/acs.biomac.5c01271 (PMC12606623; doi:10.1021/acs.biomac.5c01271)
Supplement: Supplementary file 1 [file bm5c01271_si_001.pdf]

Supporting information

Residue-Based Thermogravimetric Analysis: A Novel Method to Quantify Carboxylate Group Modification in Macromolecules

*Christos Leliopoulos, Hamidreza Mokhtari, Shima Tavakoli, Oommen P. Varghese**

Translational Chemical Biology Laboratory, Division of Macromolecular Chemistry,

Department of Chemistry-Ångstrom Laboratory, Uppsala University, Uppsala SE75121, Sweden

Supplementary text & equations

The extent of dissociation can be estimated from the Henderson–Hasselbalch relationship:

$$pH \; = \; pK_a \; + \; \log_{10}\frac{[A^-]}{[HA]} \qquad \text{(Eq. S1)}$$

For a monoprotic acid the degree of ionization,

$$\frac{[A^-]}{[HA] \; + \; [A^-]} = \frac{10^{pH \, - \, pK_a}}{1 + 10^{pH \, - \, pK_a}} \qquad \text{(Eq. S2)}$$

which can be rearranged to determine the ionization of the carboxylic acids groups as follows:

$$Ionization \; (\%) = \frac{10^{pH \, - \, pK_a}}{1 + 10^{pH \, - \, pK_a}} \times \; 100 \qquad \text{(Eq. S3)}$$

Where pH is the pH of the solution, pK_a is the acid dissociation constant of HA, $[A^-]$ concentration of conjugate base, $[HA]$ concentration of an acid and $Ionization\;(\%)$ is the percentage of the acid that is ionized in the solution.

Universal equation for DoM:

$$DoM \; (\%) = \frac{R_{sample}}{R_{control}} \; \times \; 100 \qquad \text{(Eq. S4)}$$

Where R_{sample} is the residue ratio/percent of the sample (for example modified HA), and the $R_{control}$ is the ratio/percent of the control (for example HA-unmodified)

The equation used to calculate the theoretical residue of sodium acetate is the one below:

$$R_{\mathrm{Th}} = \frac{M_{\mathrm{Na_2CO_3}}}{2 \; \times \; M_{\mathrm{C_2H_3NaO_2}}} \qquad \text{(Eq. S5)}$$

$$R_{\mathrm{Th}}(\%) = \frac{105.9888}{2 \; \times \; 82.0343} \; \times \; 100$$

$$R_{\mathrm{Th}}(\%) = 64.6\%$$

Where R_{Th} is the theoretical ratio and $R_{\mathrm{Th}}(\%)$ is the theoretical percentage of the residue, $M_{\mathrm{Na_2CO_3}}$ is the molar mass of sodium carbonate and $M_{\mathrm{C_2H_3NaO_2}}$ is the molar mass of sodium acetate.

Figure S1

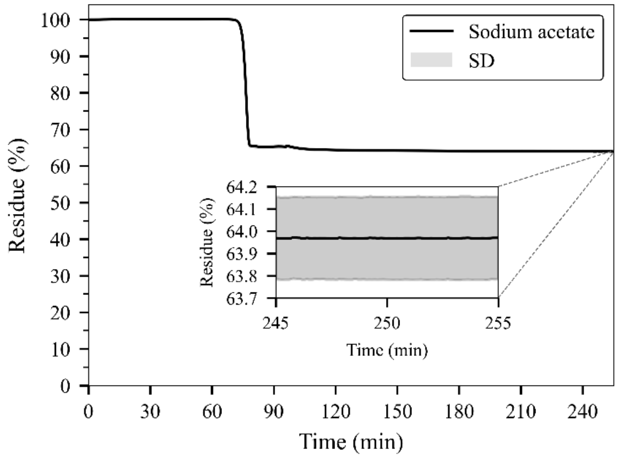

Figure S1. TGA residue profile of CH_3COONa (n=3) under the same experimental conditions as the main samples. The expected theoretical residue for Na_2CO_3 formation is 64.6%. The measured experimental residue was 63.97% ± 0.18%, which is approximately 1% lower than the theoretical value. This difference is attributed to the initial purity (99%) of the CH_3COONa used.

Figure S2

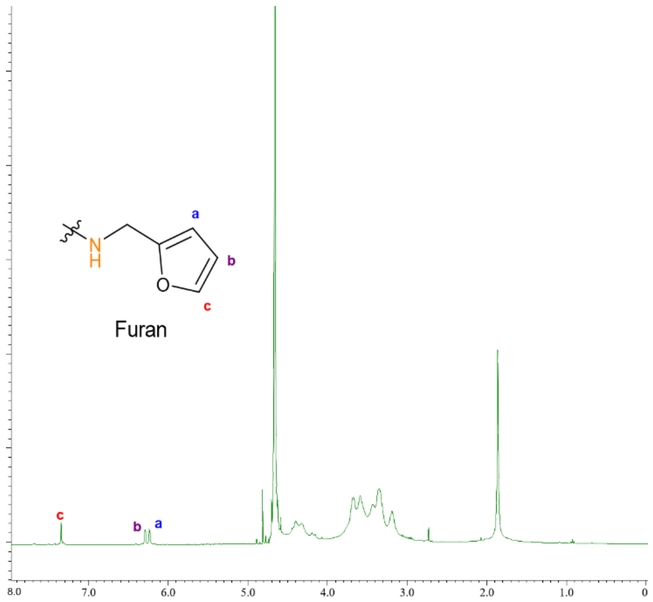

Figure S2. ^{1}H NMR spectrum of the HA-Furan. The inset structure shows the furan moiety (protons a–c), color-matched to the corresponding resonances at 6.3–7.4 ppm in the spectrum. Integration of these diagnostic signals against the native N-acetyl methyl singlet at 1.98 ppm (internal reference) was used to quantify the DoM of the carbohydrate.

Figure S3

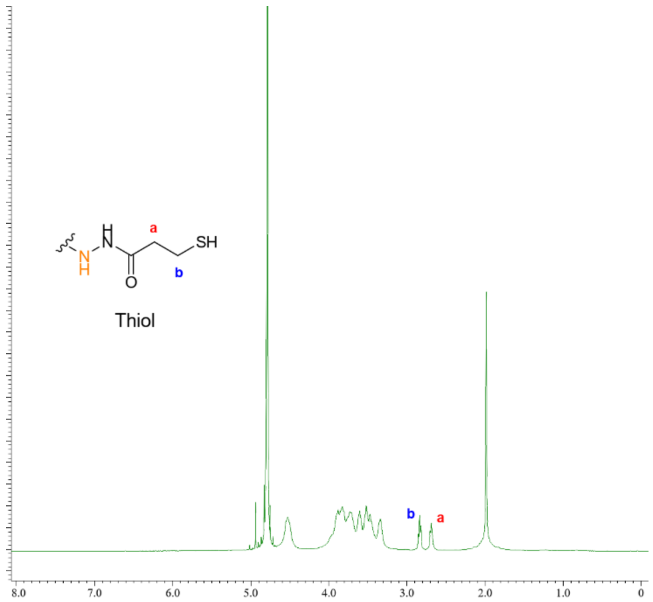

Figure S3. ^{1}H NMR spectrum of the HA-Thiol. The inset structure shows the thiol moiety (protons a and b), color-matched to the corresponding resonances at 2.83 and 2.68 ppm in the spectrum. Integration of these diagnostic signals against the native N-acetyl methyl singlet at 1.98 ppm (internal reference) was used to quantify the DoM of the carbohydrate.

Figure S4

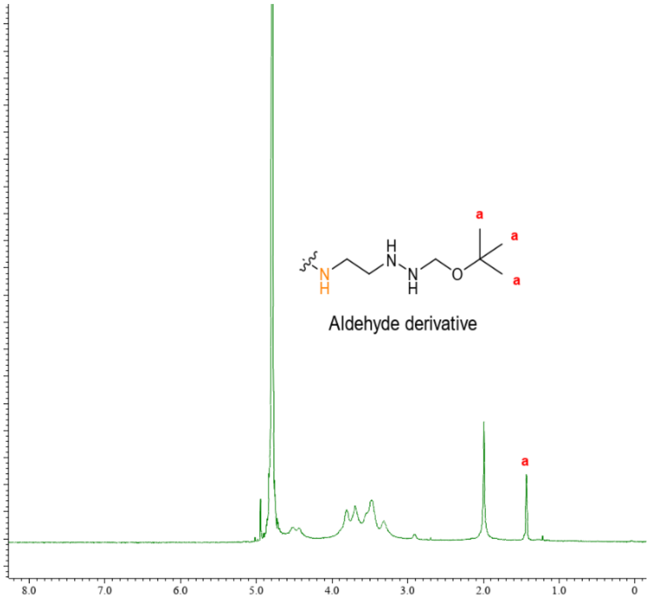

Figure S4. ^{1}H NMR spectrum of the HA-Ald. The inset structure shows the aldehyde moiety that was further modified with tert-butyl carbazate (protons a), color-matched to the corresponding resonances at 1.42 ppm in the spectrum. Integration of these diagnostic signals against the native N-acetyl methyl singlet at 1.98 ppm (internal reference) was used to quantify the DoM of the carbohydrate.

Figure S5

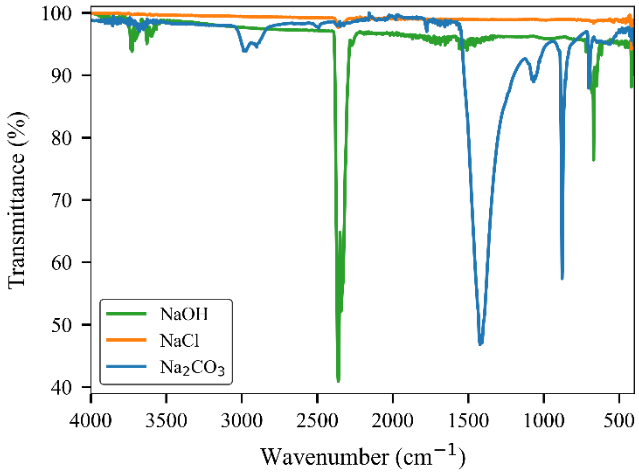

Figure S5. FT-IR transmittance spectra of NaOH, NaCl, and Na_2CO_3 (4000–400 cm). Na_2CO_3 shows characteristic carbonate bands (~1490–1410, ~880, ~680 cm^{-1}), NaOH displays weak H_2O/OH features (~3400 and ~1650 cm^{-1}).

Table S1. Ionization percentage of hyaluronic acid (pka = 3.2) at different pH values

pH	Ionization (%)	pH	Ionization (%)	pH	Ionization (%)
**3**	38.68632	**5.1**	98.75673	**7.2**	99.99
**3.1**	44.26884	**5.2**	99.0099	**7.3**	99.99206
**3.2**	50	**5.3**	99.21193	**7.4**	99.99369
**3.3**	55.73116	**5.4**	99.373	**7.5**	99.99499
**3.4**	61.31368	**5.5**	99.50131	**7.6**	99.99602
**3.5**	66.61394	**5.6**	99.60347	**7.7**	99.99684
**3.6**	71.52528	**5.7**	99.68477	**7.8**	99.99749
**3.7**	75.97469	**5.8**	99.74944	**7.9**	99.998
**3.8**	79.924	**5.9**	99.80087	**8**	99.99842
**3.9**	83.36625	**6**	99.84176	**8.1**	99.99874
**4**	86.31931	**6.1**	99.87427	**8.2**	99.999
**4.1**	88.81842	**6.2**	99.9001	**8.3**	99.99921
**4.2**	90.90909	**6.3**	99.92063	**8.4**	99.99937
**4.3**	92.64124	**6.4**	99.93694	**8.5**	99.9995
**4.4**	94.06491	**6.5**	99.94991	**8.6**	99.9996
**4.5**	95.22733	**6.6**	99.96021	**8.7**	99.99968
**4.6**	96.17135	**6.7**	99.96839	**8.8**	99.99975
**4.7**	96.93466	**6.8**	99.97489	**8.9**	99.9998
**4.8**	97.54966	**6.9**	99.98005	**9**	99.99984
**4.9**	98.04377	**7**	99.98415		
**5**	98.43983	**7.1**	99.98741		

Table S2. pH value of different NaOH concentrations and the conversion to Na_2CO_3 concentration

Target pH	*NaOH (mM)*	*NaOH (mg/mL)*	*Na_2CO_3 (mg/mL)*	*NaOH (mg/10mL)*	*Na_2CO_3 (mg/10mL)*
5.6	0.000000819	0.000000033	0.000000043	0.000000328	0.000000434
6	0.000006838	0.000000274	0.000000362	0.000002735	0.000003624
6.5	0.000028460	0.000001138	0.000001508	0.000011384	0.000015084
7	0.000096838	0.000003874	0.000005132	0.000038735	0.000051324
7.5	0.000313065	0.000012523	0.000016592	0.000125226	0.000165924
8	0.000996838	0.000039874	0.000052832	0.000398735	0.000528324
8.5	0.003159115	0.000126365	0.000167433	0.001263646	0.001674331
9	0.009996838	0.000399874	0.000529832	0.003998735	0.005298324
9.5	0.031619614	0.001264785	0.001675840	0.012647846	0.016758395
10	0.099996838	0.003999874	0.005299832	0.039998735	0.052998324

Table S3. Compilation of DoM from all methods for different HA derivatives

Name	*TGA DoM (%)*	*NMR DoM (%)*	*UV-Vis (%)*
HA-Ald	9.49 ± 2.66	10.1	-
HA-Furan	15.91 ± 0.52	16	-
HA-Thiol	29.38 ± 1.41	29.7	30.1
HA-Cyano	20.5 ± 1.54	-	-

Table S4. Compilation of the starting dry mass of the TGA samples, the residue in milligram and in percent

Name	*Starting dry mass (mg)*	*Residue mass (mg)*	*Residue (%)*
NaHA 15mg	14.672	1.856	12.65
NaHA 15mg	16.142	2.021	12.52
NaHA 15mg	14.956	1.856	12.41
NaHA 20mg	19.688	2.461	12.50
NaHA 20mg	20.426	2.588	12.67
NaHA 20mg	20.558	2.580	12.55
NaHA 25mg	25.158	3.185	12.66
NaHA 25mg	24.537	3.077	12.54
NaHA 25mg	24.288	3.053	12.57
HA-Ald	20.846	2.268	10.88
HA-Ald	19.637	2.272	11.57
HA-Ald	21.141	2.408	11.39
HA-Furan	20.272	2.084	10.28
HA-Furan	19.469	2.015	10.35
HA-Furan	20.597	2.103	10.21
HA-Thiol	18.902	1.584	8.38
HA-Thiol	19.362	1.638	8.46
HA-Thiol	16.037	1.299	8.10
HA-Cyano	19.670	1.849	9.40
HA-Cyano	19.494	1.889	9.69
HA-Cyano	20.387	2.000	9.81
